# Authors’ Reply: Vaccination, payment, and COVID-19

**DOI:** 10.4178/epih.e2021100

**Published:** 2021-11-23

**Authors:** Jelena Dotlic, Vida Jeremic Stojkovic, Paul Cummins, Marija Milic, Tatjana Gazibara

**Affiliations:** 1 Institute of Epidemiology, Faculty of Medicine, University of Belgrade, Belgrade, Serbia; 2 Department of Medical Education, Icahn School of Medicine at Mount Sinai, New York, NY, USA; 3 Faculty of Medicine, University of Pristina temporarily settled in Kosovska Mitrovica, Kosovska Mitrovica, Serbia

Dear Editor,

We read Choi’s letter with great interest [1]. However, we are compelled to respond to the criticisms in it.

First, we structured our initial comment as a response to a publication [2] that debated whether the Serbian government is paying its citizens to receive the coronavirus disease 2019 (COVID-19) vaccine. Our point was to explain that it is a mistake to characterize the actions of the Serbian government as payments as opposed to financial relief [3]. While perceptions of financial incentives can depend on numerous factors, including the manner in which they are offered [4,5], if they are not payments, then the ethical questions about paying for vaccination should be set aside. These cash transfers are either payments or financial relief; people’s perceptions of them cannot change that.

This first objection does not respond to our claim that these are not payments. Moreover, the amount of 25 euros (or any amount whatsoever) would not likely distort the perceptions of a person who categorically objects to vaccination. Moreover, this specific amount of financial relief would not materially change one’s socioeconomic status in Serbia, which is classified as an upper-middle-income country according to the World Bank categorization of worlds’ economies. Fears of post-vaccination adverse effects and potential harm can be balanced by evidence-based data, and it is not likely that this fear would be easily eliminated by offering (any amount) of money. However, it could be the “final push” for people who were hesitant and uncertain about vaccination and contribute to a change in their point of view by motivating them to seek additional information and ultimately accept vaccination. One could argue that financial incentives are a less intrusive option for increasing vaccination coverage than alternatives like punishment and movement restrictions. This is consistent with the public health consensus that the least intrusive means should be used to achieve public health goals.

Second, we reject the characterization of our explanation as a straw man fallacy because we are not attributing an artificially weak argument to people. Instead, we are objecting to the use of a term that has specific connotations related to research ethics in the context of discussing a public health ethics question. The passage cited from Jecker [4] conflates undue influence (i.e., offering excessive and unjustifiable reward to secure compliance) and coercion (i.e., convincing a person to act contrary to his or her free will through force or threats). However, to call cash transfer incentives for vaccination coercive is inaccurate given the definition of coercion. The recent public health measures in Austria, involving lockdown for non-vaccinated people [6], do represent, in fact, a genuine case of coercion. To claim that something is coercive because it feels coercive (and how is that established?) is to commit the same mistake as made in objection 1.

Third, we agree that assessing the appropriateness of a measure relative to the situation is necessary. All measures to increase vaccination coverage stemmed from the efforts to achieve optimum herd immunity as soon as possible. Therefore, financial incentives are offered as a form of a one-time reward to those who contribute to the protection of the health of the population and help to advance and develop the community. However, we do not agree that financial incentives for vaccination conflict with the necessity to identify reasons for vaccine hesitancy. The appropriateness of immunization as public health strategy has never been considered problematic. Whether or not immunization should be compulsory is indeed a matter of ethical assessment. Nevertheless, financial incentives are not part of that debate.

The main goal of research about people’s reasons underlying vaccine hesitancy is to adjust public health campaigns to address those concerns and persuade them to eventually receive the vaccine. In the meantime, given that such research may take some time to complete, public health authorities should put all their efforts into increasing vaccination rates, while balancing the value of respect for individual autonomy. Financial incentives are just one of many public health measures. Although data in the literature showed that financial incentives could indeed increase the vaccination rate [7], the long-term effects of financial and all other measures on increasing vaccine acceptance remain unclear and insufficiently explored.

Finally, we agree that a short essay is insufficient to discuss such a complex topic. The word limit, typical of medical journals, compelled us to articulate our reasoning in a concise manner. We plan to elaborate our analysis for future publications.
